# Unraveling the interplay between mesenchymal stem cells, gut microbiota, and systemic sclerosis: therapeutic implications

**DOI:** 10.1128/spectrum.01576-24

**Published:** 2025-04-24

**Authors:** Lili Zhang, Hui Wang, Lu Zhao, Jin Zhang, Wenchang Sun, Jinjin Chu, Haobin Zhao, Chunjuan Yang, Shushan Yan, Xiaohua Chen, Donghua Xu

**Affiliations:** 1 Medical Research Center, Weifang People’s Hospital, Shandong Second Medical University 372527 , Weifang, China; 2 Department of Rheumatology and Immunology, Weifang People’s Hospital, Shandong Second Medical University 372527 , Weifang, China; 3 Department of Gastrointestinal and Anal Diseases Surgery, the Affiliated Hospital, Shandong Second Medical University 372527 , Weifang, China; 4 Department of Microbiology and Immunology, Tulane University School of Medicine 12255, New Orleans, Louisiana, USA; 5 Department of Nuclear Medicine, Weifang People's Hospital, Shandong Second Medical University 372527 , Weifang, China; Tainan Hospital, Ministry of Health and Welfare, Tainan, Taiwan

**Keywords:** mesenchymal stem cells, systemic sclerosis, metabolism, 2bRAD sequence, gut microbiota

## Abstract

Systemic sclerosis (SSc) is an autoimmune disease with progressive fibrotic disorders in multiple organs. Mesenchymal stem cells (MSCs) have shown great potential in treating SSc, but the exact regulatory mechanism is not fully understood. In this study, we used human umbilical cord-derived MSCs (hUC-MSCs) to treat SSc mice induced by bleomycin. The gut microbiota composition and predicted functions were analyzed using 2bRAD sequencing of fecal samples from control, SSc, and MSCs-treated mice. Treatment with MSCs improved the bleomycin-induced SSc mice, characterized by significantly reduced collagen deposition and dermal thickness. The gut microbiota of SSc mice exhibited lower species evenness and was clearly separated from the control mice based on beta diversity. MSC treatment led to a significant reduction of conditionally pathogenic bacteria enriched in SSc, including *Akkermansia muciniphila* and *Parasutterella excrementihominis*. Conversely, the relative abundance of butyrate-producing bacteria, such as *Roseburia*, *Butyricicoccus porcorum*, and *Gemmiger formicilis*, was notably increased in MSCs-treated SSc mice. Additionally, the functional analysis revealed that MSCs intervention effectively enhanced sulfur metabolism, tryptophan metabolism, citrate cycle, RNA polymerase, and beta-lactam resistance. In summary, the findings in the present study have suggested the close association between gut microbiota and metabolic dysbiosis in mice with SSc. The administration of MSCs has been shown to regulate the disrupted metabolic pathways in SSc mice, thus restoring the normal function of the gut microbiota. This study provides valuable insights into the specific gut microbiota and metabolic pathways involved in the efficacy of MSC treatment, thereby proposing a novel therapeutic strategy for SSc.

**IMPORTANCE:**

Human umbilical cord-derived mesenchymal stem cells (HUC‑MSCs) demonstrate efficacy in alleviating skin thickening and collagen deposition in systemic sclerosis (SSc) mice, which also regulate the gut microbiota composition and function. Specifically, MSC intervention leads to a notable increase in butyrate-producing bacteria, a decrease in *Akkermansia muciniphila* and *Parasutterella excrementihominis*, and a reversal of the dysregulated microbial function in SSc mice. These findings underscore the potential significance of gut microbiota in the therapeutic effects of MSCs in SSc.

## INTRODUCTION

Systemic sclerosis (SSc) is a rare immune-mediated autoimmune disease characterized by vasculopathy, immune dysregulation, and fibrosis of skins and organs (1). The global incidence of SSc is 18.87 per 100,000, with a higher prevalence in females (2). The pathogenesis of SSc has not yet been well understood, although some factors are well documented to be associated with SSc pathogenesis, such as genetic and environmental factors, endothelial damage, vasculitis, immune imbalance, and fibroblast dysfunction (3). Currently, there are no effective treatments for SSc (4). The main clinical therapeutic goal is to achieve symptomatic relief. Consequently, there is an urgent need for the exploration of innovative therapies for SSc.

In the last decade, mesenchymal stem cells (MSCs) have garnered significant attention due to self-renewal and multidirectional differentiation potentials. The therapeutic potential of MSCs has also been widely investigated in autoimmune diseases (5), neurological diseases (6), and endocrine disorders (7). Clinical trials have shown that MSC treatment can effectively alleviate skin ulcers, necrosis, and pain in patients with SSc (8). Nevertheless, the molecular mechanisms underlying the beneficial effects of MSCs in SSc remain poorly understood. The gut microbiota is often referred to as the “second genome” of the human body, influencing the integrity of the intestinal barrier, inflammatory status, immune metabolism, and microenvironmental homeostasis (9). Gastrointestinal (GI) involvement is a common early manifestation of SSc, affecting over 90% of patients. Most SSc patients with severe GI disease have a poor prognosis and high mortality (10). Dysbiosis of the gut microbiota has been observed in patients with SSc (11–14). It has been demonstrated that gut microbiota interventions, such as dietary changes, probiotics, and fecal microbiota transplantation, have emerged as promising therapies for SSc patients (15). However, interventions targeting gut microbiota may need to be combined with additional therapeutic approaches due to the heterogeneous manifestations of SSc patients. Previous studies have suggested that MSC treatment may modulate the gut microbiota in various diseases, including inflammatory bowel disease (16–18), acute liver injury (19), and pulmonary hypertension (20). How MSCs interact with gut microbiota and the mechanism underlying MSCs-mediated improvement in SSc is not yet known.

In the current study, 2bRAD sequencing technology was applied to explore the role of MSCs in regulating the structure and function of gut microbiota in bleomycin-induced SSc mice. This work is aimed at providing insights into novel therapeutic strategies of SSc based on the disease-specific gut microbiota.

## MATERIALS AND METHODS

### Isolation, culture, and determination of MSCs

MSCs were isolated and cultured following a previously published protocol (21). Briefly, Wharton’s jelly was stripped from the umbilical cord, which was cut into 1 mm^3^ pieces. Tissue blocks were then incubated in complete DMEM/F12 medium added with 1% penicillin and 100 U/mL streptomycin (Gibco, NY, USA). Cells adherent to the plate were detached after approximately 2 weeks using 0.25% trypsin-ethylenediaminetetraacetic acid (Gibco, NY, USA) and transferred to new flasks for further expansion. MSCs were determined by flow cytometry analysis of the specific markers before use, including CD90, CD44, CD73, CD34, CD45, and HLA-DR (eBioscience, California, USA) on MSCs. In subsequent experiments, MSCs from passages 3 to 5 were used. The current research was approved by the Ethics Committee of the First Affiliated Hospital of Shandong Second Medical University (KYLL20230306-1). Prior to study participation, all participants signed an informed consent form.

### Animal experiment

The animal study was carried out under the supervision of Shandong Second Medical University’s Animal Ethics Committee (2023SDL156). Fifteen female C57BL/6J mice aged 6 weeks and free of specific pathogens were purchased from the Medical Experimental Animal Center of Shandong Second Medical University for experiments. Our study exclusively examined female mice because the disease is more common in women. The animal room was maintained at 25°C under 50% relative humidity and a 12 h light and darkness cycle. Following a 1-week acclimatization period, 10 mice were randomly selected to receive a daily injection of 100 µL bleomycin (10 mg/kg) at a single site on the shaved back for a duration of 28 days. The remaining five mice served as controls (CON) and received an equivalent volume of phosphate-buffered saline (PBS). Subsequently, 10 bleomycin-treated mice were randomly selected for either the SSc model group (SSc) or the MSC-treated group (MSC). A volume of 200 µL PBS containing 5 × 10^5^ MSCs was injected into the tail vein of each mouse in the MSC group bi-weekly, a total of two injections per mouse. Control and model mice received an equal volume of PBS. All mice were anesthetized and sacrificed for further estimation 2 weeks later.

### Histological analysis

The skins of each mouse were removed and fixed in 4% paraformaldehyde. Paraffin-embedded sections (4 µm) were used for hematoxylin and eosin (H&E) and Masson’s trichrome assays based on the protocols (Solarbio, China). Tissue sections were scanned by use of the Motic Easyscan digital slide scanner (Motic, USA) and further analyzed with Motic DSAssistant software. In order to assess the severity of skin sclerosis, dermal thickness between the epidermal-dermal junction and the dermo-fat junction was measured. Mean dermal thickness was calculated based on measurements taken at more than 30 randomly selected points from each section.

### Collection and DNA extraction of fecal samples

Fresh fecal samples were collected from mice before they were sacrificed and stored at −80°C freezer. DNAs were isolated from feces using the Agencourt AMPure XP kit (Beckman Coulter, Brea, CA). DNA extraction was performed using a NanoDrop 2000 UV-visible spectrophotometer (Thermo Scientific, Wilmington, DE) to estimate the concentration and purity. DNA quality was verified by electrophoresis on 1% agarose gels.

### Library construction and sequencing

The 2bRAD-M library was constructed according to the original protocol published previously (22) with minor adjustments. DNA samples were firstly digested by the BcgI enzyme at 37°C for 3 h. DNA fragments were ligated to adaptors, amplified, and resolved on the polyacrylamide gel. DNA of approximately 100 bp was further eluted from gel bands. After PCR, the products were purified and sequenced using Illumina Nova PE150 in the OE Biotech Company in Qingdao, China. All sequencing data were deposited in the NCBI SRA database (accession number: PRJNA1073456).

### Identification of species-specific markers

A total of 173,165 microbial genomes from the NCBI RefSeq database were downloaded for sequence alignment. Subsequently, using Perl scripts, each type 2B restriction enzyme was used to establish an extensive 2bRAD database. A unique identifier was assigned to each set of 2bRAD tags. To identify the unique 2bRAD tags within a genome without overlap, all 2bRAD tags were compared. Finally, species-specific 2bRAD markers were developed based on those specific 2bRAD tags. The database contained 26,163 microbial species with 2bRAD tags that were theoretically unique. All sequenced tags were mapped against the 2bRAD marker database to identify microbial species. The threshold G score was set at 5 in order to prevent false positives (23). For each species, the average read coverage of all 2bRAD markers, namely the relative abundance, was determined for subsequent analyses.

### Statistical analysis

Statistical analysis was conducted using the software packages R (version 3.2.0) and GraphPad Prism (version 8.3.0). R software was used for principal coordinate analysis (PCoA), visualization of species abundance spectra, and functional abundance spectra. Analysis of similarity (ANOSIM) was used to perform PCoA statistical analysis based on the equidistant matrix. Specific species and functions were identified by the linear discriminant analysis effect size (LEfSe). Continuous variables were presented as $\overset{-}{x}$ ± s and analyzed using one-way analysis of variance (ANOVA) or the Kruskal-Wallis (K-W) test. For the comparison between pairwise data, the least significant difference multiple-comparison post hoc test was applied in the one-way ANOVA, while Dunn’s multiple-comparison post hoc test was performed with the Kruskal-Wallis test. *P* < 0.05 is considered statistically significant.

## RESULTS

### Identification of MSCs

**Fig 1:**
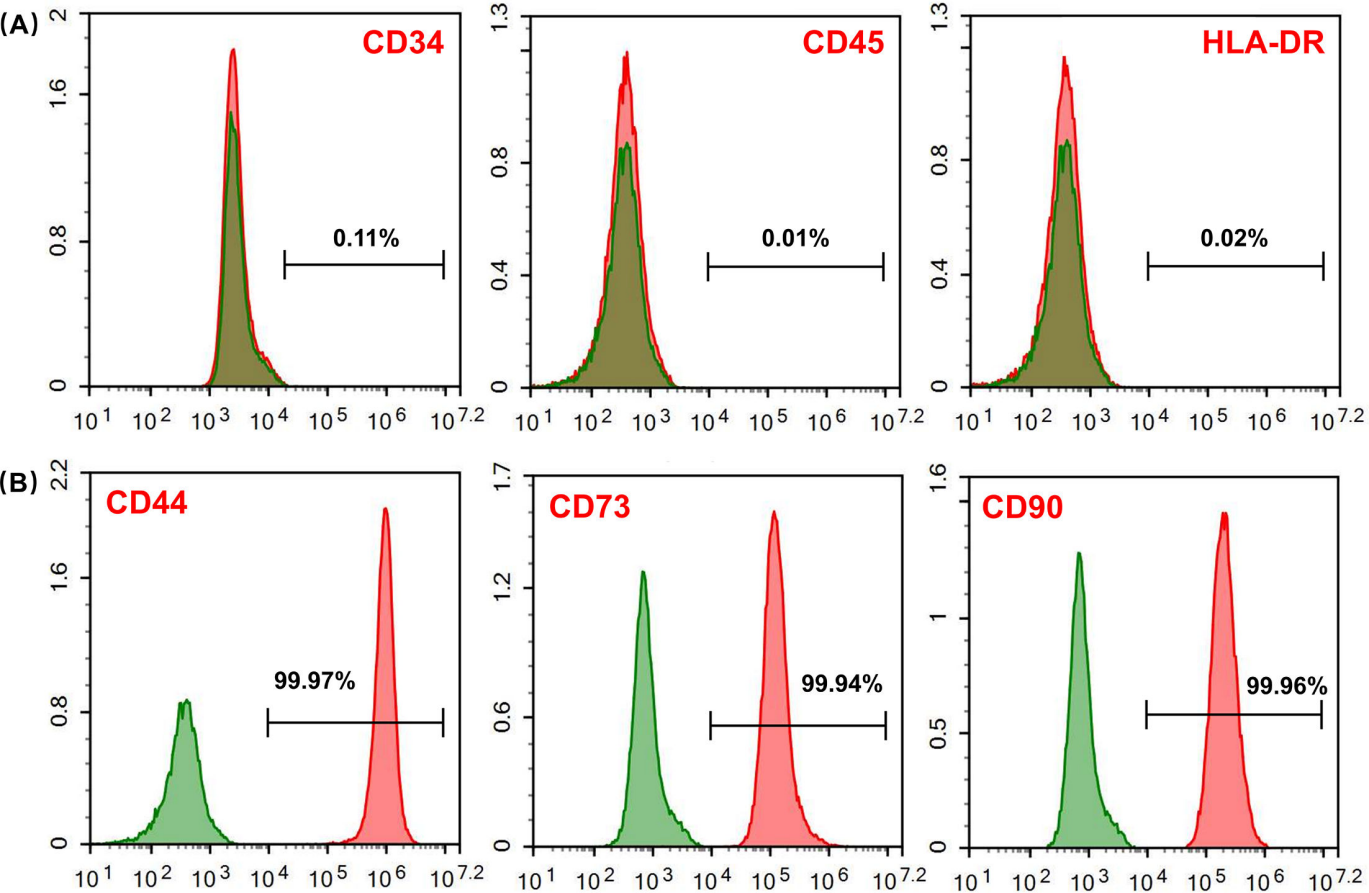
Phenotype identification of MSCs. (**A**) Negative surface markers (CD34, CD45, and HLA-DR) identification of MSCs. (**B**) Positive surface markers (CD44, CD73, and CD90) identification of MSCs.

After 5–7 days of incubation in cell culture medium, MSCs crawled out from human umbilical cord tissues, showing spindle shape and sparse distribution around the tissue. Surface markers of MSCs were detected using flow cytometry. MSCs were negative with CD45, CD34, and HLA-DR (Fig. 1A), but positive with mesenchymal markers CD90, CD73, and CD44 (Fig. 1B).

### Human umbilical cord-derived MSCs alleviated SSc mice induced by bleomycin

**Fig 2:**
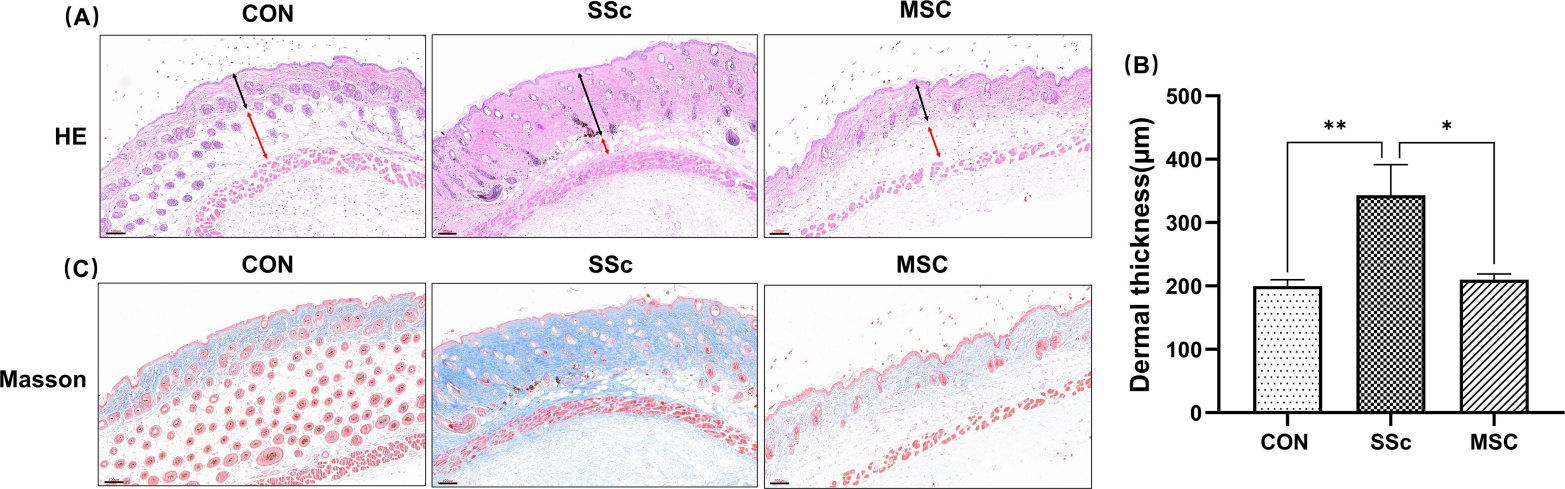
hUC-MSCs alleviated bleomycin-induced skin sclerosis in mice. (**A**) Representative images of H&E staining of the skin from mice of CON, SSc, and MSC groups (100×). The dermis is marked by the black bidirectional arrow. The subcutaneous fat layer is marked by the red bidirectional arrow. Scale bar = 100 µm. (**B**) Measuring the thickness of the dermis in the black skin of mice. Values were determined in at least 30 random points from each section. CON *n* = 5; SSc *n* = 4; MSC *n* = 4; data are shown as mean ± standard error of mean (SEM). *, *P* < 0.05; **, *P* < 0.01. (**C**) Representative images of Masson-Trichrome staining of the skin from mice of CON, SSc, and MSC groups (100×). The collagen fibers are dyed blue. Scale bar = 100 µm.

The primary manifestations of SSc mice included skin thickening, collagen fibers increase, and disappearance of subcutaneous fat. In this study, skins from SSc mice exhibited a reduced and even disappeared subcutaneous fat layer (Fig. 2A), increased thickness of the dermal layer (Fig. 2B) and collagen fibers (Fig. 2C), suggesting a successfully established SSc mice model induced by bleomycin. The administration of human umbilical cord-derived MSCs (hUC-MSCs) significantly helped to attenuate the dermal thickness, collagen depositions, and subcutaneous fat thinning of SSc mice. These findings suggested that hUC-MSCs might hold considerable potential in the treatment of SSc.

### Gut microbiota composition between CON, SSc, and MSC groups

**Fig 3:**
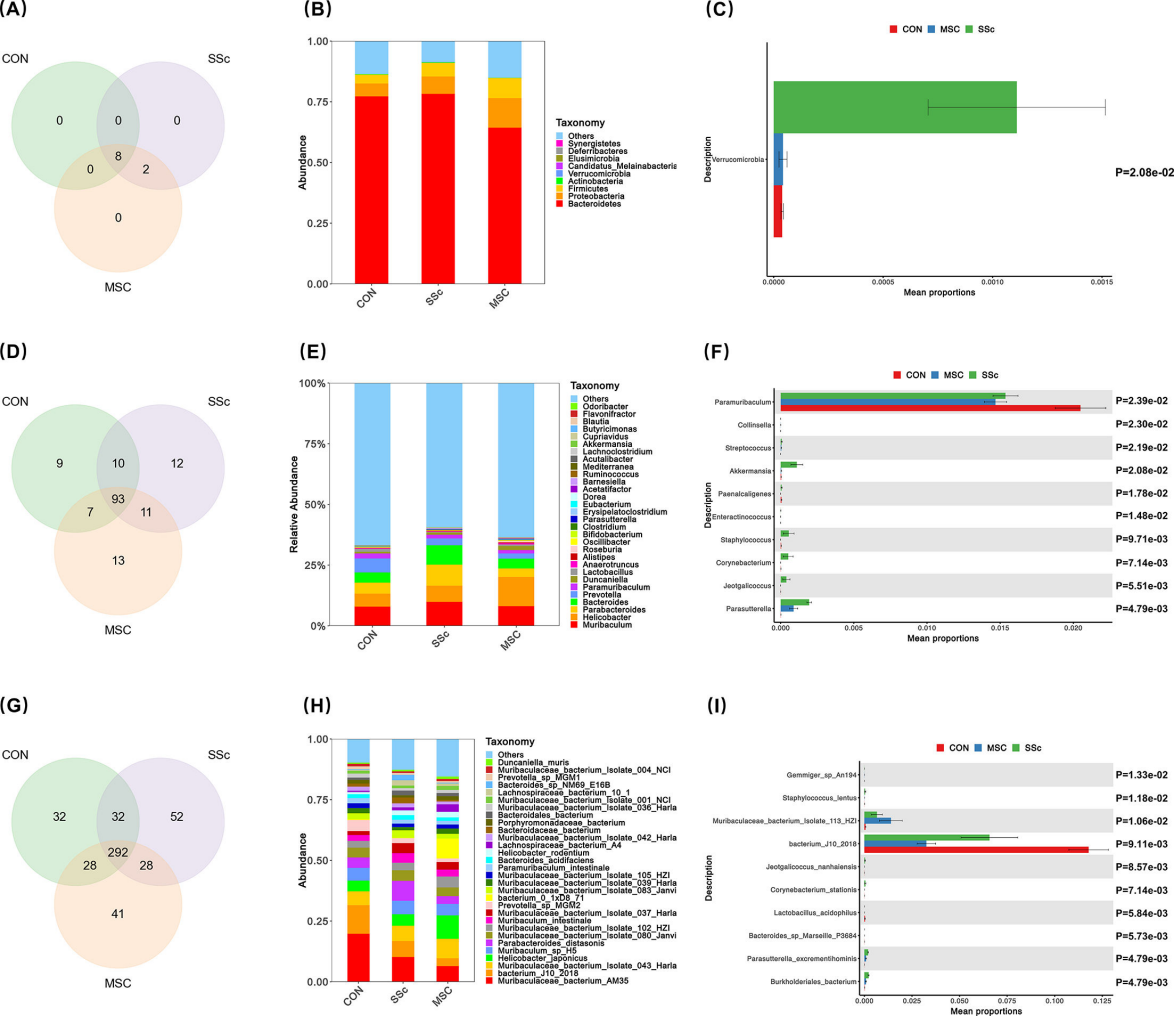
Composition of gut microbiota in the CON, SSc, and MSC groups. (**A–C**) Species number, gut microbiota composition, and the top 10 species at the phylum level. (**D–F**) Species number, gut microbiota composition, and the top 10 species at the genus level. (**G–I**) Species number, gut microbiota composition, and the top 10 species at the species levels.

To elucidate whether MSCs alleviated SSc via gut microbiota, fecal samples of mice from CON, SSc, and MSC groups were sequenced by 2bRAD-M. Compared with conventional methods, such as 16S rRNA amplicon sequencing and metagenomic sequencing, 2bRAD-M offers highly accurate species-level profiles even in challenging sample types characterized by partial degradation, low biomass, and high host contamination (23). The relative abundance of species at each taxonomic level was calculated according to the method described above. Nine phyla were identified at the phylum level, with seven phyla common and two phyla (*Elusimicrobia* and *Synergistetes*) shared between the SSc and MSC groups (Fig. 3A). The predominant phyla, namely *Bacteroidetes*, *Proteobacteria*, and *Firmicutes,* collectively accounted for over 99% of the total bacteria (Fig. 3B). Specifically, *Bacteroidetes* was the most abundant phyla, accounting for 77.22% of the CON group, 78.27% of the SSc group, and 64.33% of the MSC group. Furthermore, *Verrucomicrobia* were significantly more abundant in the SSc group than in the CON and MSC groups (*P* < 0.05) (Fig. 3C). At the genus level, unclassified bacteria predominated, comprising over 50% of the bacterial composition in each group. Of the identified 154 bacterial genera, 93 genera were present in all three groups, with nine unique genera in the CON group, 12 in the SSc group, and 13 in the MSC group (Fig. 3D). *Muribaculum*, *Helicobacter*, *Parabacteroides*, *Bacteroides*, and *Prevotella* were the dominant genera, representing >80% of the identified bacteria (Fig. 3E). In the CON and SSc groups, the proportion of *Muribaculum* was the highest, accounting for 7.94% and 9.86%, respectively, while *Helicobacter* was predominant (11.96%) in the MSC group. Fifteen genera exhibited statistically significant differences among the three groups, the top 10 genera of which were presented in Fig. 3F, including *Parasutterella*, *Jeotgalicoccus*, *Corynebacterium*, *Staphylococcus*, *Enteractinococcus*, *Paenalcaligenes*, *Akkermansia*, *Streptococcus*, *Collinsella*, and *Paramuribaculum*. At the species level, there were 292 common species in the three groups, while 32 species were unique to the CON group, 52 species to the SSc group, and 41 to the MSC group (Fig. 3G). Analysis of the top 30 species of gut microbiota in the three groups revealed significant differences in species distribution (Fig. 3H). Additionally, the K-W test identified 48 species showing statistical differences among the three groups, with the top 10 species displayed in Fig. 3I. These data suggest that both disease status and MSCs intervention exert effects on the composition of gut microbiota.

### Diversity of gut microbiota between groups

**Fig 4:**
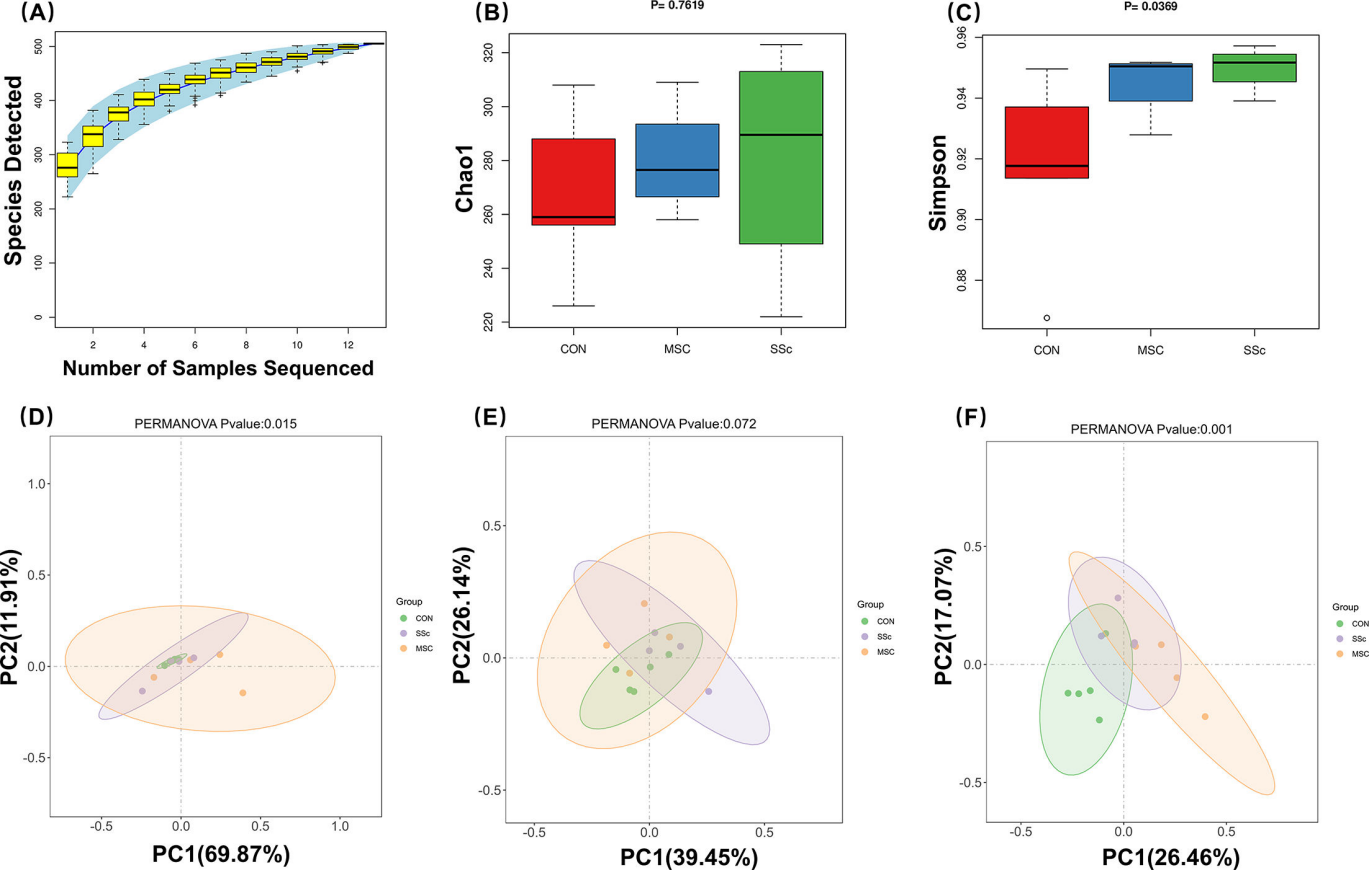
The diversity of the gut microbiota among CON, SSc, and MSC mice. (**A**) The species accumulation curve of species numbers among the three groups. (**B and C**) Chao1 and Simpson indexes of gut microbiota α diversity between groups. (**D–F**) PCoA analysis of gut microbiota β diversity between groups at the phylum, genus, and species levels.

The species accumulation curve is an effective tool for evaluating and predicting the increase in species richness with sample size, commonly used to estimate sample adequacy and community abundance. A flattening curve indicates sufficient sample size for sequencing. As shown in Fig. 4A, the species accumulation curve plateaued when the number of samples reached 13, suggesting saturation of detected species and adequate sample size for diversity analysis. We used Chao1 and Simpson indices to assess the α diversity of the gut microbiota. The Chao1 index was applied to estimate species richness, while the Simpson index represented species evenness. In terms of richness, the three groups did not differ significantly (*P* = 0.762) (Fig. 4B). However, species evenness in the SSc and MSC groups was significantly lower than that in the CON group (*P* = 0.037) (Fig. 4C). The PCoA analysis using the Binary-Jaccard distance matrix was conducted to evaluate gut microbiota β diversity at the phylum (Fig. 4D), genus (Fig. 4E), and species (Fig. 4F) levels. Based on comparisons of ANOSIM of the gut microbiota β diversity, significant differences were found at phylum and species levels but not at the genera level (phylum, *P* = 0.015; genus, *P* = 0.072; species, *P* = 0.001). These findings imply that disease status and MSCs intervention may have an impact on gut microbiota diversity.

### Specific species of gut microbiota

**Fig 5:**
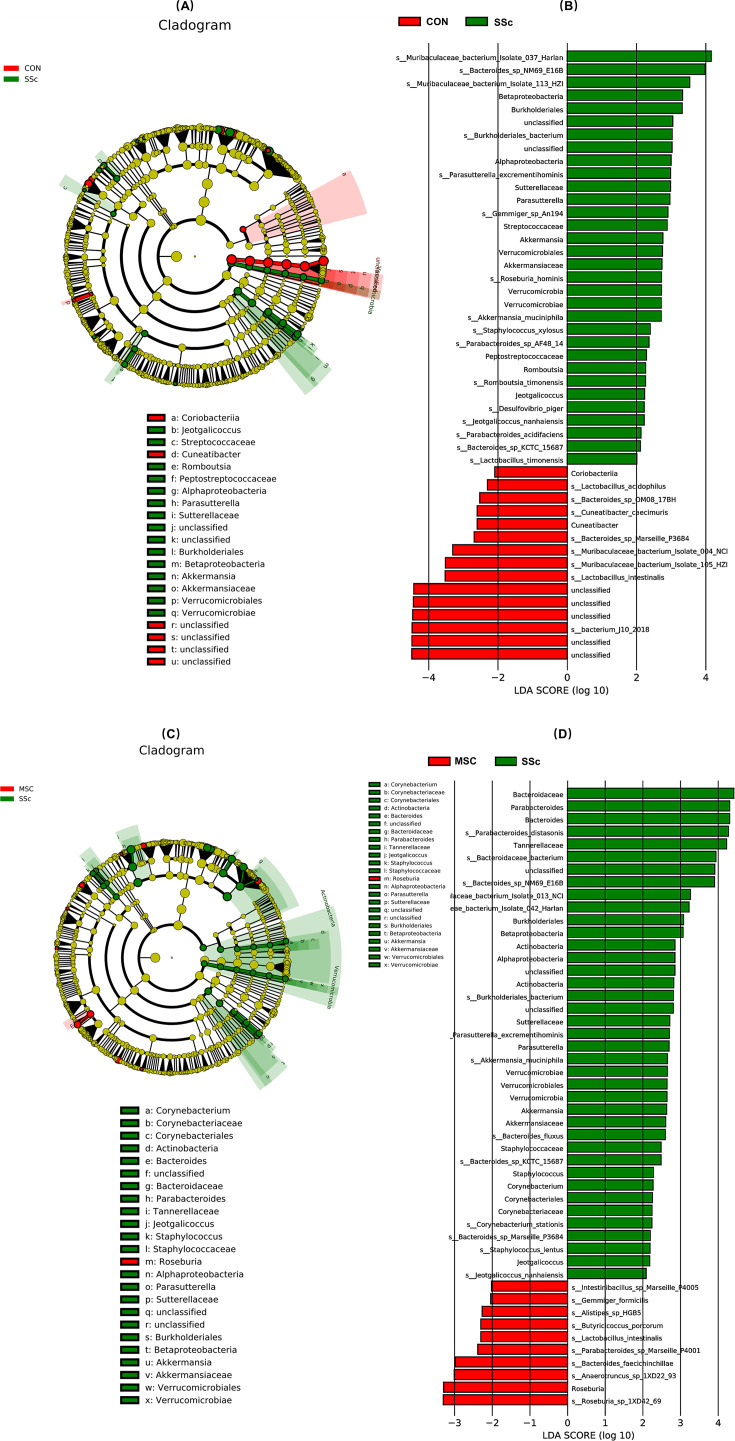
Specific species of gut microbiota between groups. (**A**) Annotated branch diagram of gut microbiota-specific species between CON and SSc mice. (**B**) Taxa with differential abundance between CON and SSc mice analyzed with LefSe. (**C**) Annotated branch diagram of gut microbiota-specific species between SSc and MSC mice. (**D**) Taxa with differential abundance between SSc and MSC mice analyzed with LefSe. Only species with an LDA of >2 are shown.

We used LEfSe analysis to screen different biomarkers of gut microbiota associated with SSc and MSC treatment. To explore the effects of SSc induced by bleomycin on gut microbiome, the different bacterial biomarkers between the CON group and the SSc group were analyzed. Taxonomic cladograms revealed the dominant gut microbiota species across both groups at the phylum to genus level (Fig. 5A). Based on linear discriminant analysis (LDA) scores above 2.0, we identified 40 distinguishing taxa between the CON and MSC groups (Fig. 5B). Compared to the CON Group, *Muribaculaceae bacterium* Isolate-037 (Harlan), *Bacteroides* sp. NM69_E16B, *M. bacterium* Isolate-113 (HZI), *Burkholderiales bacterium*, *Parasutterella excrementihominis*, *Gemmiger* sp. An194, *Roseburia hominis*, *Akkermansia muciniphila*, *Staphylococcus xylosus*, *Parabacteroides* sp. AF48-14, *Romboutsia timonensis*, *Desulfovibrio piger*, *Jeotgalicoccus nanhaiensis*, *Parabacteroides acidifaciens*, *Bacteroides* sp. KCTC 15687, and *Lactobacillus timonensis* were significantly enriched in the SSc group at the species level. Notably, the increased abundance of *B. bacterium* and *P. excrementihominis* was associated with the increase of *Betaproteobacteria* and *Burkholderiales*, with subsequent increases in *P. excrementihominis* further contributing to the rise of *Sutterellaceae* and *Parasutterella*. Additionally, the enrichment of *A. muciniphila* was related to increased levels of *Verrucomicrobiae*, *Verrucomicrobiales*, *Akkermansiaceae*, and *Akkermansia*. However, the SSc group had a lower relative abundance of *Ligula intestinalis*, *M. bacterium* Isolate-105 (HZI), *M. bacterium* Isolate-004 (NCI), *Bacteroides* sp. Marseille-P3684, *Cuneatibacter*, *Cuneatibacter caecimuris, Bacteroides* sp. OM08-17BH, *Lactobacillus acidophilus*, and *Coriobacteriia*, with the exception of unclassified bacteria.

To further estimate the effect of MSC treatment on the gut microbiota of SSc mice, the specific species between the SSc group and MSC group were assessed (Fig. 5C and D). After treatment with MSCs, the notably decreased bacteria were *Bacteroidaceae*, *Parabacteroides*, *Bacteroides*, *Parabacteroides distasonis*, and *Tannerellaceae,* with the LDA value above 4. Additionally, many of the bacteria enriched in the SSc group were notably diminished post-treatment. These included the classes *Betaproteobacteria*, *Alphaproteobacteria*, and *Verrucomicrobiae*; the orders *Burkholderiales* and *Verrucomicrobiales*; the families *Sutterellaceae* and *Akkermansiaceae*; the genera *Parasutterella*, *Akkermansia*, and *Jeotgalicoccus*; and the species *Bacteroides* sp. NM69_E16B, *B. bacterium*, *P. excrementihominis*, *A. muciniphila*, *Bacteroides* sp. KCTC 15687, *J. nanhaiensi*. Moreover, a noteworthy finding was the significant increase in butyrate-producing bacteria and probiotics (*Roseburia* sp. 1XD42-69, *Roseburia*, *Anaerotruncus* sp. 1XD22-93, *Butyricicoccus porcorum*, *Alistipes* sp. HGB5, *Gemmiger formicilis*, and *L. intestinalis*) in the MSC group. Taken together, MSC treatment demonstrated the capability to reduce the overgrowth of microflora enriched in the SSc mice while promoting the number of beneficial bacteria.

### Gut microbiota function analysis

**Fig 6:**
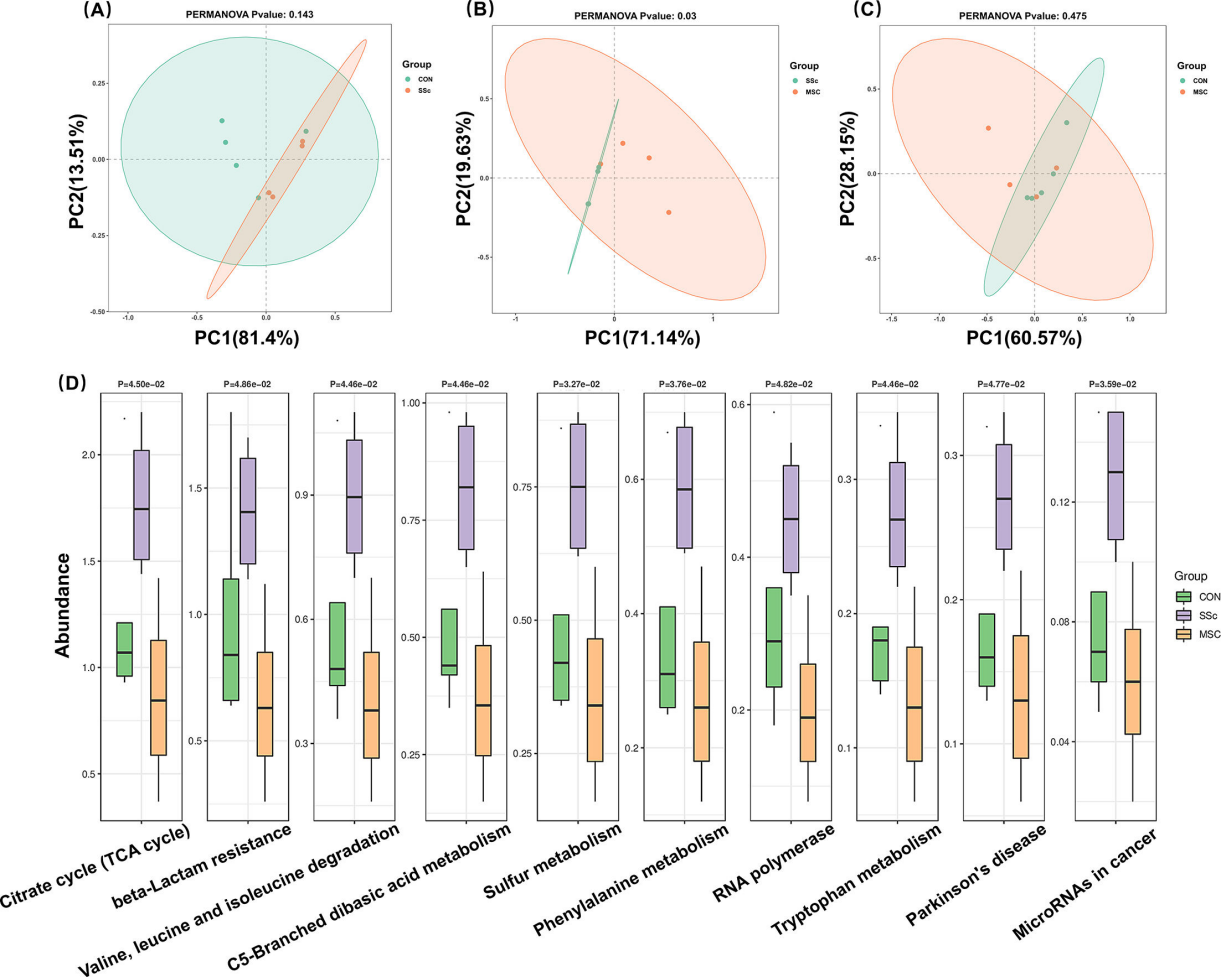
Function of gut microbiota predicted by PICRUSt2 in the CON, SSc, and MSC groups. (**A**) PCoA of gut microbiota function analysis. (**B**) PCoA of gut microbiota function analysis. (**C**) PCoA of gut microbiota function analysis. (**D**) Gut microbiota function enriched analysis.

Gut microbiota function was further predicted at the KEGG L3 level by the use of PICRUSt2 software. The function of gut microbiota had no obvious difference between the CON and SSC groups (Fig. 6A) and the CON and MSC groups (Fig. 6B) according to the PCoA based on the Binary-Jaccard distance matrix. However, MSC treatment altered gut microbiota function compared to the SSC group (Fig. 6C). Based on the K-W test between the three groups, a total of 10 specific pathways, including citrate cycle, beta-lactam resistance, valine, leucine and isoleucine degradation, C5-branched dibasic acid metabolism, sulfur metabolism, phenylalanine metabolism, RNA polymerase, tryptophan metabolism, Parkinson’s disease, and microRNAs in cancer, were all enriched in the SSC group (Fig. 6D). MSC treatment effectively reduced these functional levels of gut microbiota to levels comparable to those in the control group. Accordingly, MSC therapy not only contributes to the restoration of gut microbiota structure but also corrects the dysregulated gut microbiota function.

## DISCUSSION

Increasing number of studies have underscored the significance of gut microbiota in the therapeutic application of MSCs for various diseases. However, specific alterations and contributions of gut microbiota in the treatment of SSc using MSCs remain largely unknown. In this study, we aimed to investigate the modifying effects of MSC treatment on gut microbiota in SSc model mice induced by bleomycin. The 2bRAD high-throughput sequencing technology was used to examine the composition of gut microbiota in fecal samples obtained from SSc and MSCs-treated mice. The findings of the study revealed that the intervention of MSCs did not significantly impact the diversity of gut microbiota in SSc mice, whereas MSC treatment resulted in a decrease in the evenness of gut microbiota and a notable alteration in the overall structure. In particular, the intervention of MSCs notably diminished the presence of species that were enriched in the SSc group while increasing the abundance of bacteria producing butyrate. Furthermore, MSC treatment rescued certain gut microbiota functions, which were significantly elevated in SSc mice. This study has provided novel insights into the mechanism by which MSCs contribute to the therapeutic potentials for SSc, highlighting the interaction of gut microbiota with MSC treatment.

The association between the α diversity of gut microbiota and SSc remains unclear, with conflicting findings across different studies. While some investigations have shown no significant differences in the α diversity of gut microbiota between SSc patients and healthy controls (13, 24–26), others have presented inconsistent results. Tang et al. have found no significant alterations in the α diversity of the microflora in the bleomycin-induced SSc mice (27). The analysis of data collected from 106 SSc patients in Sweden revealed contrasting results, with a statistically significant increase in the Shannon index among SSc patients compared to healthy controls (28). Patrone’s study revealed no significant alteration in gut flora α diversity among SSc patients, but a substantial 10-fold decrease was observed in SSc compared to healthy controls (12). Similarly, a significant reduction in gut microbiota richness was demonstrated among progressive SSc patients compared to early-stage SSc patients, suggesting a possible association between disease status and changes in the α diversity of gut microbiota (29). It is worth noting that decreased α diversity within gut microbiota is common in inflammatory and immune-mediated diseases, such as inflammatory bowel disease (30, 31), systemic lupus erythematosus (32), and rheumatoid arthritis (33). Reduced α diversity has been found to be strongly linked to the progression of rheumatoid arthritis. Our study using a bleomycin-induced SSc mouse model showed no significant difference in microflora richness but a notable decrease in evenness, potentially associated with the severity of SSc in mice. In animal models of inflammatory bowel disease (16, 17) and pulmonary hypertension (20), treatment with MSCs has been shown to restore the diminished intestinal α diversity. However, in an animal model of acute liver injury, MSC intervention had no effect on the α diversity of microflora (19). In our study, the intervention of MSCs did not alter the reduced evenness in gut microbiota induced by bleomycin. Consequently, further investigations are necessary to ascertain the impact of MSCs intervention on the diversity of the gut microbiota.

In clinical samples obtained from SSc patients and healthy controls, the dominant gut microbiota phyla were *Firmicutes*, *Bacteroides*, and *Proteobacteria*. It was notable that in SSc patients' intestines, *Firmicutes* and *Bacteroides* were significantly decreased compared to healthy controls (25–27, 29). In SSc animal models, *Bacteroides* exhibited the highest proportion among bacteria, while the abundance of *Firmicutes* was significantly increased following bleomycin induction (27). In our SSc mouse model, the three prevailing phyla were *Bacteroides*, *Proteobacteria*, and *Firmicutes*. Although no significant distinction was observed among these three phyla in control and model mice, *Firmicutes* displayed a tendency toward augmentation in the model group. This suggests that consortia derived from *Firmicutes* may play a pivotal role in SSc progression, although further research is needed to confirm their contributions.

There is a notable reduction in beneficial commensal microorganisms and an elevation in potentially pathogenic bacteria within gut microbiota in SSc (15). In our study, increased conditional pathogenic proinflammatory bacteria was demonstrated in the gut microbiota from SSc mice, including *β-Proteobacteria*, *Burkholderiales*, *α-Proteobacteria*, and *D. piger*. Besides, a decrease in commensal probiotics in gut microbiota was observed in SSc, such as *L. intestinalis*, *C. caecimuris*, and *L. acidophilus*. Furthermore, several strains of gut microbiota exhibited high abundance in SSc mice. Among these strains, *A. muciniphila* has emerged as a promising contender for the next generation of probiotics, which belongs to the *Verrucomicrobia* phylum and is specialized in the degradation of intestinal mucin (34). However, excessive accumulation of *A. muciniphila* in compromised intestinal barriers may alter mucin degradation, leading to further deterioration of intestinal integrity (35), heightened inflammation (36), and an elevated likelihood of tumor formation (37). Additionally, *A. muciniphila* enrichment is recurrent and widespread within the intestinal tract of individuals diagnosed with multiple sclerosis (38–40), indicating its involvement in immune regulation. Nevertheless, the precise regulatory mechanism of *A. muciniphila* remains unclear. Hence, elucidating the role and mechanism of *A. muciniphila* in the pathogenesis of SSc may offer novel strategies for the intervention of SSc. *P. excrementihominis,* a prevalent taxon in *Parasutterella*, is a singular species representing the genus in human specimens. Its relative abundance is linked to various health outcomes, although the precise function remains unknown (41). In the context of endocrine and metabolic disorders, such as nonalcoholic fatty liver disease (42), prenatal stress (43), and social fear (44), a decrease in *P. excrementihominis* abundance may potentially contribute to the preservation of host health. Conversely, in elderly patients with inflammatory bowel disease, where intestinal function is compromised, *P. excrementihominis* is found to be significantly elevated in the intestines, exacerbating the deterioration of intestinal health. *P. excrementihominis* exhibits notable enrichment within the gastrointestinal tracts of patients with inflammatory bowel disease (45) and elderly individuals who engage in insufficient physical activity (46). In a murine model of depression, colonization by *P. excrementihominis* intensifies depressive behaviors, yet the administration of ginkgo biloba extract effectively diminishes the abundance and alleviates symptoms of the disease (47). Notably, our study suggests that *P. excrementihominis* is elevated in SSc. Therefore, we hypothesize that the increase of *P. excrementihominis* may potentially be linked to the compromised gastrointestinal function resulting from SSc. However, further investigation is warranted to illustrate this issue.

*Lactobacillus*, a widely employed commercial probiotic, is often utilized to bolster gastrointestinal function and promote host well-being (48). Nevertheless, the elevated levels of *Lactobacillus* in the gut of SSc patients have prompted researchers to question its role (15). In our SSc model, the enrichment of *L. timonensis* was observed in SSc mice, while some strains such as *L. intestinalis* and *L. acidophilus* were significantly diminished. These findings imply a potential systematic bias in human-derived samples. It’s noteworthy that a substantial quantity of glucocorticoid is administered during SSc treatment (49). Also, the abundance of *Lactobacillus* bacteria within the genus is increased due to the utilization of high-dose dexamethasone (50). The impact of medications on the prevalence of *Lactobacillus* has been validated in diabetes, where antidiabetic metformin administration notably enhances the abundance of *Lactobacillus* (51). Therefore, it’s crucial to select clinical samples when investigating the specific species that exert pivotal effects on SSc progression, particularly considering some confounding factors that can significantly affect the composition of gut microbiota, such as diet and medication.

Short-chain fatty acids, primarily including butyrate, acetate, and propionate, are the key byproducts from bacterial fermentation of dietary starch and fibers (52). These metabolites play significant roles in the gastrointestinal tract, directly influencing the metabolic activities and epigenetic state of host T cells, thereby contributing to immune regulation in type 1 diabetes, multiple sclerosis, rheumatoid arthritis, and other autoimmune disorders (53). *Faecalibacterium prausnitzii*, a typical butyric acid-producing bacteria, has been found to be significantly reduced in SSc (11, 24, 26), suggesting that butyric acid-producing bacteria may have a crucial role in inhibiting the progression of SSc. Our study has revealed that treatment with MSCs not only reduced the abundance of species enriched in SSc mice but also increased butyric acid-producing bacteria, such as *Roseburia*, *B. porcorum*, and *G. formicilis*. Previous research has documented the ability of butyrate to facilitate the differentiation of MSCs into various tissue cells, thereby enhancing the regenerative potential and tissue repair capacity of MSCs (54, 55). Hence, the therapeutic efficacy of MSCs in SSc may be associated with the butyrate-producing bacteria. However, the underlying mechanism needs to be clarified in future studies.

In addition to alterations in the composition of gut microbiota, significant differences in function were observed between CON, SSc, and MSC groups. In the present study, sulfur metabolism was significantly augmented in SSc mice. It was potentially linked to *Desulfovibrio piger* proliferation, leading to hydrogen sulfide overproduction and intestinal barrier disruption (56). Hydrogen sulfide in the intestine protects the integrity of the intestinal barrier (57). Kynurenine, an intermediate of tryptophan metabolism, is found to accumulate excessively in the plasma of patients with SSc (58, 59). Overexpression of kynurenine leads to the dysregulation of Treg cells mediated by the cascade of proinflammatory cytokines (60), disrupting the immune microenvironment of SSc and aggravating disease progression (61). Additionally, the heightened tryptophan metabolism in gut microbiota may impact the progression of SSc by metabolites involved in the regulation of the host immune system. Succinate is an important component of the TCA cycles, exhibiting increased levels in the plasma of patients with SSc (62). It plays a critical role in mediating inflammation and fibrosis in intestinal epithelial cells by interacting with the receptor GPR91 (63). Thus, the enhanced functionality of the TCA cycle in SSc mice may contribute to the progression of SSc by facilitating tissue fibrosis via metabolites. In the immunological categorization of SSc, cases with positive anti-RNA polymerase antibodies usually exhibit diffuse SSc with a relatively unfavorable prognosis and an elevated risk of cancer (64). In patients with localized SSc, initial cutaneous lesions can be managed through the administration of antibiotics, such as penicillin (65). Conversely, SSc mice exhibit heightened resistance to β-lactam antibiotics, which consequently impacts the efficacy of drug-based interventions. In conclusion, the administration of bleomycin in mice may augment the progression of SSc by enhancing the functionality of the aforementioned gut microbiota. MSCs intervention can inhibit the pathogenic functions of gut microflora, suggesting potential therapeutic benefits in SSc. A better understanding of the mechanisms underlying the interaction between MSCs and gut microflora and the potential benefits of MSC treatment in SSc requires further research.

In conclusion, our study highlights significant alterations in gut microbiota composition and function in SSc-model and MSCs-treated mice. These findings suggest that MSC therapy may modulate the dysregulated microbiota in SSc, improving the intestinal microecology. Nevertheless, the SSc mouse model induced by bleomycin may not fully reflect the complexities of human SSc. Some factors like diet, medication, disease stage, and environment might influence the disease outcomes and gut microbiota. Moreover, the difference in gut microbiota between human and mouse underscores the need for clinical investigations to further confirm these findings.

## Data Availability

Data are available from the corresponding author upon request.
